# Supplementary material for: Detection of Echinococcus multilocularis in coyotes in Washington State, USA highlights need for increased wildlife surveillance
Source: PLoS Negl Trop Dis. 2026 Mar 24;20(3):e0013502. doi: 10.1371/journal.pntd.0013502 (PMC13012483; doi:10.1371/journal.pntd.0013502)
Supplement: S1 Table — (DOCX) [file pntd.0013502.s001.docx]

| **Species** | **Sample type** | **Source (as received by our lab)** | **Host** |
| --- | --- | --- | --- |
| *Taenia taeniaeformis* | Positive control sample | Extracted DNA (unknown stage) | Unknown |
| *Mesocestoides* sp. | Positive control sample | Extracted DNA from adult | Dog |
| *Mesocestoides tetrathyridium* | Positive control sample | Extracted DNA from cyst | Rat |
| *Dipylidium caninum* | Positive control sample | Extracted DNA from adult | Dog |
| *Spirometra* sp. | Positive control sample | Extracted DNA from adult | Cat |
| *Taenia hydatigena* | Positive control sample | Extracted DNA (unknown stage) | Unknown |
| *Echinococcus granulosus sensu stricto* | Positive control sample | Extracted DNA from cyst | Moose |
| *Echinococcus canadensis sensu lato* | Positive control sample | Extracted DNA from fecal float | Wolf |
| *Taenia pisiformis* | Positive control sample | Tissue sample | Coyote |
| *Taenia pisiformis* | Positive control sample | Extracted DNA from fecal float | Dog |
| *Echinococcus multilocularis* | Positive control sample | Tissue sample from adult | Coyote |
| *Taenia ovis* | GenBank sequence | JX134125.1 | Unknown |
| *Taenia krabbei* | GenBank sequence | MT226756.1 | Unknown |
| *Taenia asiatica* | GenBank sequence | AB031356.1 | Unknown |
| *Taenia martis* | GenBank sequence | JX415820.1, LT837855.1 | Unknown |
| *Taenia serialis* | GenBank sequences | DQ104236.1, DQ104238.1, KF414739.1, EU219546.1, LC085644.1 | Unknown |
| *Taenia multiceps* | GenBank sequence | GQ228818.1 | Unknown |
| *Taenia solium* | GenBank sequence | AB031357.1 | Unknown |
| *Taenia polyacantha* | GenBank sequence | DQ408419.1 | Unknown |
| *Mesocestoides lineatus* | GenBank sequence | AB787552.1, AB787553.1, JF268553.1, EF567417.1, L49450.1 | Unknown |
| *Mesocestoides canislagopodis* | GenBank sequence | KT232151.1 | Unknown |
| *Mesocestoides litteratus* | GenBank sequence | MN505202.1, JN088186.1, MN505203.1 | Unknown |
| *Mesocestoides lineatus* | GenBank sequence | EF567417.1, AB787552.1, AB787553.1 | Unknown |
| *Taenia crassiceps* | GenBank sequence | AB031358.1 | Unknown |
