# Supplementary material for: Detection of Echinococcus multilocularis in coyotes in Washington State, USA highlights need for increased wildlife surveillance
Source: PLoS Negl Trop Dis. 2026 Mar 24;20(3):e0013502. doi: 10.1371/journal.pntd.0013502 (PMC13012483; doi:10.1371/journal.pntd.0013502)
Supplement: S2 Table — The last row indicates the proportion of the number of samples in which the species was detected in that method (i.e., fecal swab or fecal matter) over the number of samples in which the species was detected using intestinal swabs, using our sequence read cutoff of 1000. For example, T. pisiformis was detected in 13 intestinal swabs, but only detected in 5 fecal swabs (with a read cutoff of 1000); therefore, detection success for this method was 0.38. (DOCX) [file pntd.0013502.s002.docx]

|  | *Taenia pisiformis* | |  | *Echinococcus multilocularis* | | |
| --- | --- | --- | --- | --- | --- | --- |
| **Carcass ID** | **Intestine swab** | **Fecal swab** | **Fecal matter (200 mg)** | **Intestine swab** | **Fecal swab** | **Fecal matter (200 mg)** |
| YH016 | 182512 | 0 | 0 | 2447 | 0 | 0 |
| YH035 | 124372 | 2035 | 150363 | 0 | 0 | 0 |
| YH044 | 125873 | 13443 | 99 | 1257 | 7124 | 811 |
| YH046 | 60289 | 2854 | 70041 | 0 | 0 | 0 |
| YH060 | 0 | 0 | 0 | 0 | 0 | 0 |
| YH062 | 5241 | 0 | 0 | 246344 | 152 | 1629 |
| YH066 | 125423 | 507 | 0 | 0 | 0 | 0 |
| YH100 | 172713 | 0 | 175 | 0 | 0 | 0 |
| YH126 | 12136 | 0 | 2945 | 201985 | 58526 | 0 |
| YH140 | 78892 | 277091 | 365655 | 0 | 0 | 0 |
| YH208 | 127163 | 0 | 0 | 0 | 0 | 0 |
| YH212 | 82900 | 0 | 0 | 0 | 0 | 0 |
| YH216 | 12037 | 4186 | 1316 | 147369 | 338323 | 96494 |
| YH223 | 90849 | 0 | 0 | 139 | 0 | 0 |
| Avg read count (>0) | | 50,019 | 84,361 |  | 101,031 | 32,978 |
| **Detection success** | | **0.38** | **0.38** |  | **0.66** | **0.50** |
