# Supplementary figures and images for: Detection of Echinococcus multilocularis in coyotes in Washington State, USA highlights need for increased wildlife surveillance
Source: PLoS Negl Trop Dis. 2026 Mar 24;20(3):e0013502. doi: 10.1371/journal.pntd.0013502 (PMC13012483; doi:10.1371/journal.pntd.0013502)

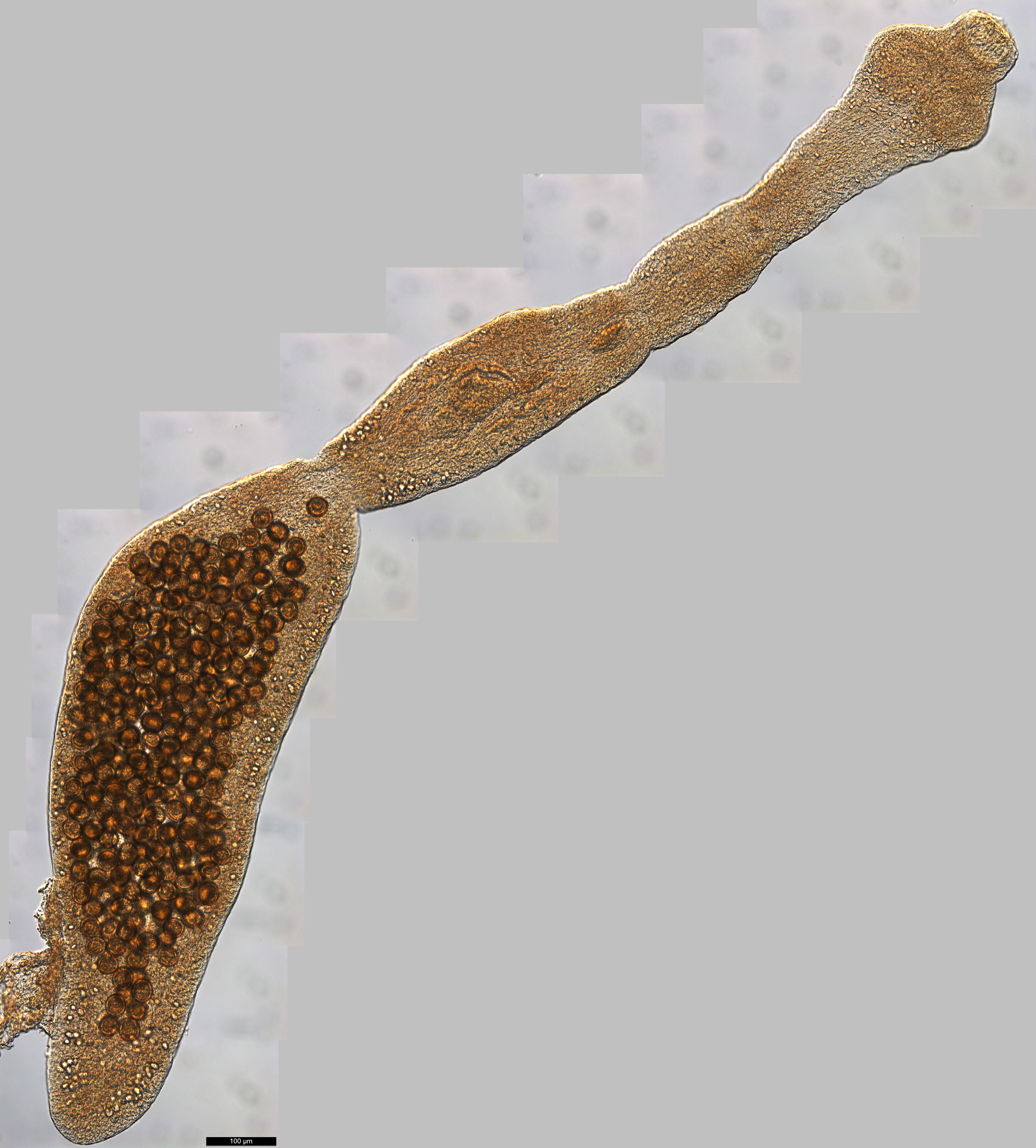

Supplement: S2 Fig — (JPG) [file pntd.0013502.s006.jpg]

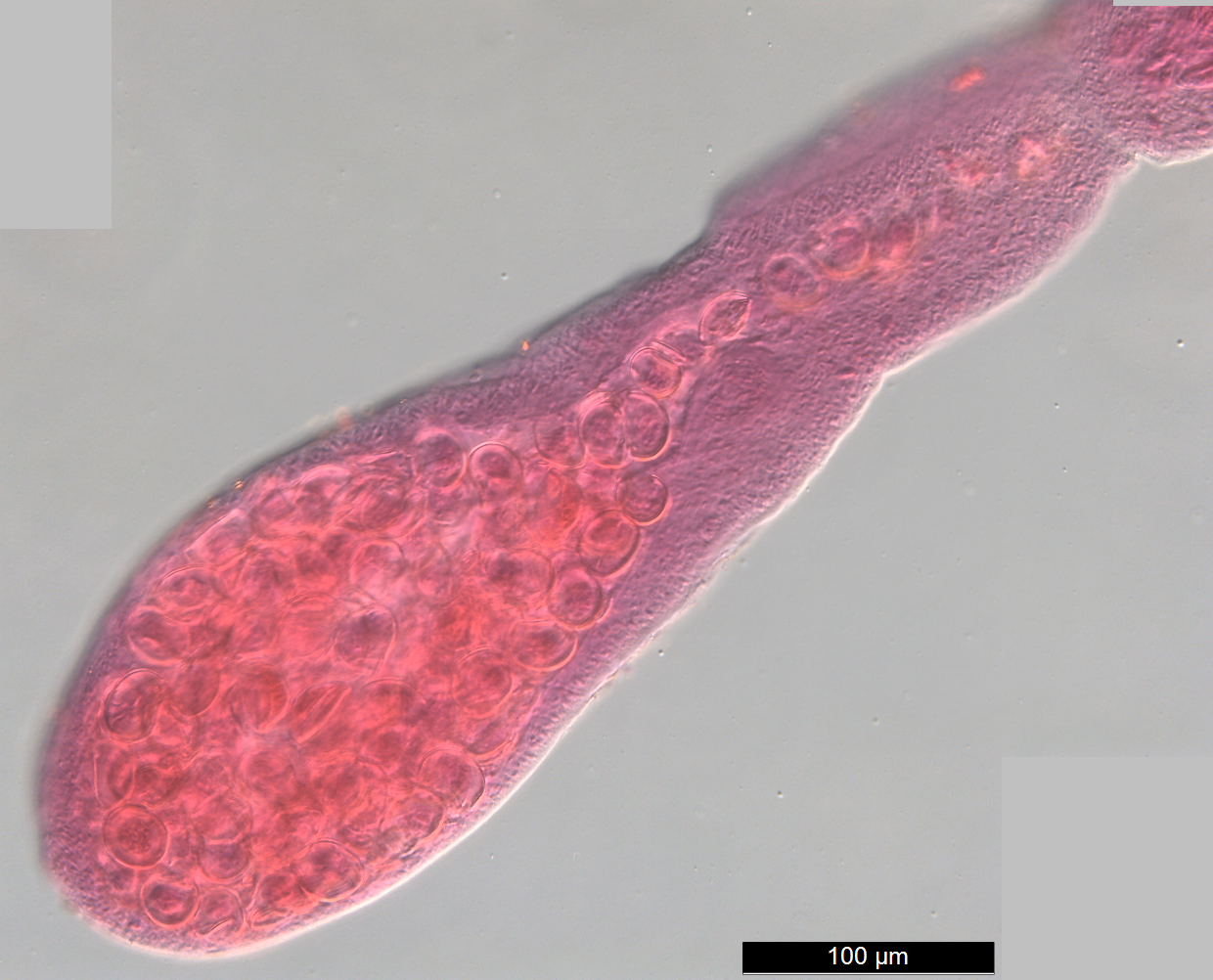

Supplement: S3 Fig — (PNG) [file pntd.0013502.s007.png]

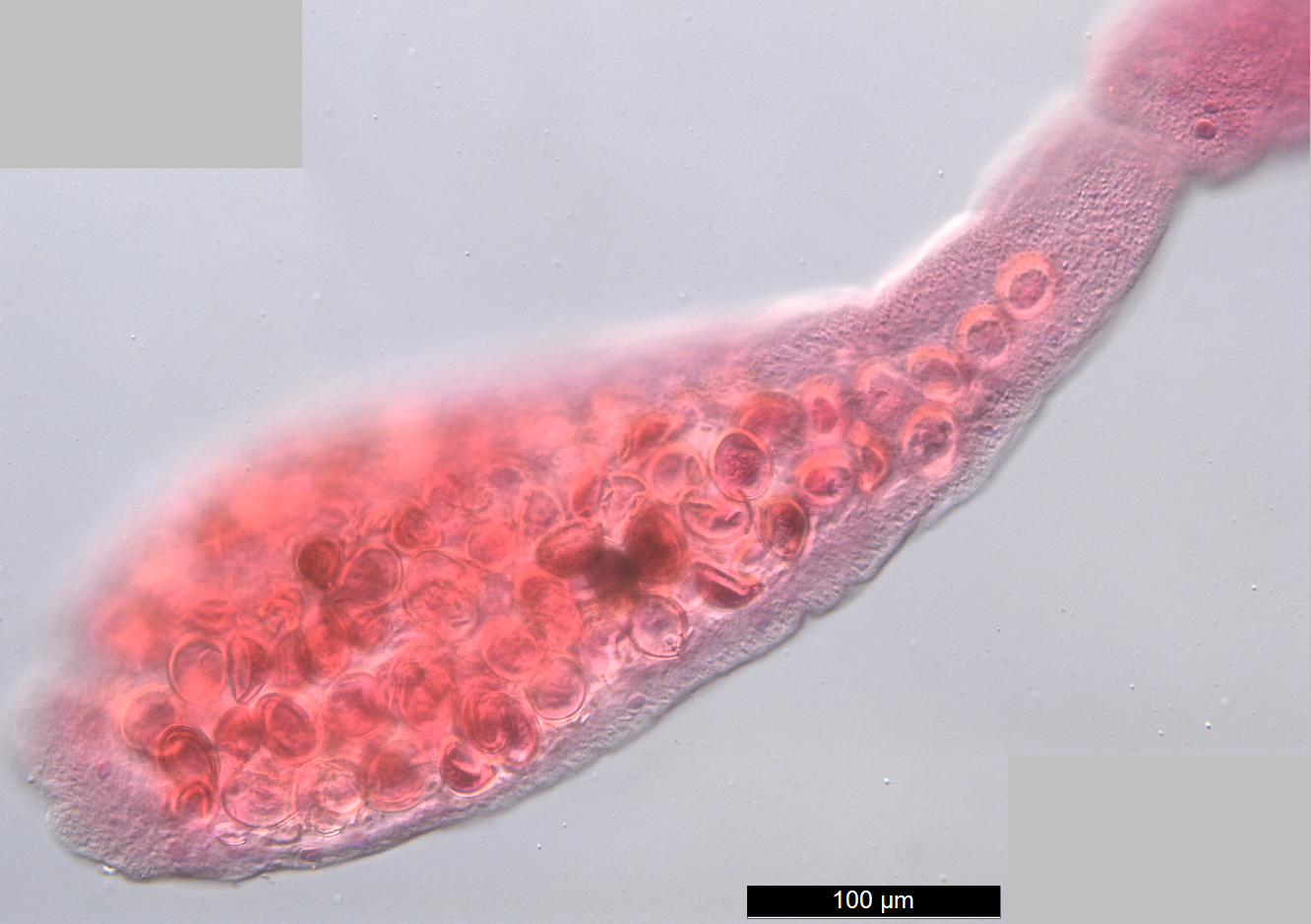

Supplement: S4 Fig — (PNG) [file pntd.0013502.s008.png]
