# Supplementary material for: Detection of Echinococcus multilocularis in coyotes in Washington State, USA highlights need for increased wildlife surveillance
Source: PLoS Negl Trop Dis. 2026 Mar 24;20(3):e0013502. doi: 10.1371/journal.pntd.0013502 (PMC13012483; doi:10.1371/journal.pntd.0013502)
Supplement: S1 Text — (DOCX) [file pntd.0013502.s009.docx]

Maps were hand-drawn using Procreate and labeled using Adobe Photoshop. We started by using the following review papers: Eckert et al. 2001 [1], Torgerson et al. 2010 [2], Davidson et al. 2012 [3], Oksanen et al. 2016 [4], Deplazes et al. 2017 [5], and Lundström-Stadelmann et al. 2025 [6]. All are peer-reviewed journal manuscripts except Eckert et al., which is a book published by the World Health Organization and the World Organisation for Animal Health. We assumed that these sources covered global reports of *E. multilocularis* across humans, domestic animals, and wildlife up until 2016 (Lundström-Stadelmann et al. was limited to human cases). We also used manuscripts cited in each of these papers when possible, as not every country discussed in the review papers had a year associated with the first report, and some maps colored entire countries rather than only regions where *E. multilocularis* was detected. Using the review papers and peer-manuscripts collected from these papers, we started drawing our map based on the first year *E. multilocularis* was detected in each country in the northern Hemisphere, as well as all states and provinces in North America (excluding Mexico). When possible, we only filled in regions with confirmed detections, rather than entire countries. We also noted differences between maps or text across all jurisdictions mentioned in these sources, and searched their cited manuscripts. For example, *E. multilocularis* had not been detected in northern France by 1999 and was therefore not included in Figure 4.3 of Eckert et al., but had been detected by 2017 and was included in Figure 1 of Deplazes et al. In this case, we found the relevant manuscript cited in Deplazes et al. [7] and used their map to inform ours.

Next, for every country in Europe and Asia, as well as every province in Canada and every state in the United States, we searched the term “echinococcus multilocularis AND (jurisdiction name)” in Google Scholar and Web of Science. For this search, we focused on papers where the authors confirmed the presence of *E. multilocularis* morphologically or molecularly, i.e. empirical studies, though we searched citations of additional review papers to find empirical studies that may not have come up in our search engine searches. We considered each paper that contained both the species name and the name of the searched jurisdiction. We referenced our list of manuscripts collected from the previous step and made any necessary changes to improve granularity and regional accuracy of our map.

For papers describing human or domestic animal cases, we read each paper to determine whether the detection was thought to be acquired in a location other than that where it was diagnosed (i.e., due to travel), and only kept papers where the patient was determined by the authors to have been infected locally. Our search was limited to peer-reviewed manuscripts, books, or book chapters in the English and French languages or that had abstracts in English or French. We may therefore have missed local case reports and other published or unpublished sources in languages other than English or French, and relied on the review sources for areas we could not confirm for ourselves. For endemic areas of Russia and China where it was difficult to find peer-reviewed empirical research in English and first detections were prior to 1990, we followed Figure 1 of Deplazes et al. 2017 and Figure 4.3 of Eckert et al. 2001.

In all cases, we used the year of collection of the confirmed sample or diagnosed patient reported in manuscripts when possible, not the publication year. When year of collection was not indicated in the manuscript, we used publication year.

All publications used in the map creation, as per the above methods, are cited below [8-92].

**References**

1. Eckert, J., M. Gemmell, F. Meslin, and Z. Pawłowski. 2001. WHO/OIE manual on echinococcosis in humans and animals: a public health problem of global concern.
2. Torgerson, P. R., A. Schweiger, P. Deplazes, M. Pohar, J. Reichen, R. W. Ammann, P. E. Tarr, N. Halkic, and B. Müllhaupt. 2008. Alveolar echinococcosis: from a deadly disease to a well-controlled infection. Relative survival and economic analysis in Switzerland over the last 35 years. Journal of hepatology 49:72–77.
3. Davidson, R. K., T. Romig, E. Jenkins, M. Tryland, and L. J. Robertson. 2012. The impact of globalisation on the distribution of Echinococcus multilocularis. Trends in parasitology 28:239–247.
4. Oksanen, A., M. Siles-Lucas, J. Karamon, A. Possenti, F. J. Conraths, T. Romig, P. Wysocki, A. Mannocci, D. Mipatrini, G. La Torre, B. Boufana, and A. Casulli. 2016. The geographical distribution and prevalence of Echinococcus multilocularis in animals in the European Union and adjacent countries: a systematic review and meta-analysis. Parasites & vectors 9:519.
5. Deplazes, P., L. Rinaldi, C. A. Alvarez Rojas, P. R. Torgerson, M. F. Harandi, T. Romig, D. Antolova, J. M. Schurer, S. Lahmar, G. Cringoli, J. Magambo, R. C. A. Thompson, and E. J. Jenkins. 2017. Global Distribution of Alveolar and Cystic Echinococcosis. Advances in parasitology 95:315–493.
6. Lundström-Stadelmann, B., A. Rostami, C. F. Frey, P. R. Torgerson, S. M. Riahi, K. Bagheri, M. Kaethner, A. Lachenmayer, G. Beldi, R. B. Gasser, and A. Hemphill. 2025. Human alveolar echinococcosis-global, regional, and national annual incidence and prevalence rates. Clinical microbiology and infection: the official publication of the European Society of Clinical Microbiology and Infectious Diseases.
7. Combes, B., S. Comte, V. Raton, F. Raoul, F. Boué, G. Umhang, S. Favier, C. Dunoyer, N. Woronoff, and P. Giraudoux. 2012. Westward spread of Echinococcus multilocularis in foxes, France, 2005-2010. Emerging infectious diseases 18:2059–2062.
8. Ahmed, H., S. Ali, M. S. Afzal, A. A. Khan, H. Raza, Z. H. Shah, and S. Simsek. 2017. Why more research needs to be done on echinococcosis in Pakistan. Infectious diseases of poverty 6:90.
9. Andreyanov, O. N. 2011. Examining Echinococcus multilocularis Infection in Some Midland Russia Predatory Animal Species. Russian Journal of Parasitology:7–11.
10. Antolová, D., V. Šnábel, J. Jarošová, S. Cavallero, S. D’Amelio, Y. Syrota, R. Rosoľanka, M. Avdičová, and M. Miterpáková. 2024. Human alveolar echinococcosis in Slovakia: Epidemiology and genetic diversity of Echinococcus multilocularis, 2000-2023. PLoS neglected tropical diseases 18:e0011876.
11. Bagrade, G., V. Šnábel, T. Romig, J. Ozoliņš, M. Hüttner, M. Miterpáková, D. Ševcová, and P. Dubinský. 2008. Echinococcus multilocularis is a frequent parasite of red foxes (Vulpes vulpes) in Latvia. Helminthologia 45:157–161.
12. Balog, T., G. Nagy, T. Halász, E. Csányi, Z. Zomborszky, and Á. Csivincsik. 2021. The occurrence of Echinococcus spp. in golden jackal (Canis aureus) in southwestern Hungary: Should we need to rethink its expansion? Parasitology international 80:102214.
13. Barabási, S. S., P. Deplazes, V. Cozma, S. Pop, C. Tivadar, I. Bogolin, and R. Popescu. 2010a. Echinococcus multilocularis confirmed in Romania. Sci Parasitol 11:89–96.
14. Beck, R., Ž. Mihaljević, R. Brezak, S. Bosnić, I. L. Janković, and P. Deplazes. 2018. First detection of Echinococcus multilocularis in Croatia. Parasitology research 117:617–621.
15. Bobić, B., T. Štajner, V. Ćirković, J. Srbljanović, O. Lijeskić, N. Bauman, and Đ. Zlatković. 2025. Human echinococcosis in the Russian Federation in the 21st century: A systematic review. Microorganisms 13:1122.
16. Borhani, M., S. Fathi, E. Darabi, F. Jalousian, S. Simsek, H. Ahmed, H. K. Kesik, S. H. Hosseini, T. Romig, M. F. Harandi, and I. Mobedi. 2021. Echinococcoses in Iran, Turkey, and Pakistan: Old diseases in the new millennium. Clinical microbiology reviews 34:e0029020.
17. Bruzinskaite, R., A. Marcinkute, K. Strupas, V. Sokolovas, P. Deplazes, A. Mathis, C. Eddi, and M. Sarkūnas. 2007. Alveolar echinococcosis, Lithuania. Emerging infectious diseases 13:1618–1619.
18. Cafiero, S. A., L. Petroni, L. Natucci, O. Tomassini, T. Romig, M. Wassermann, C. Rossi, H. C. Hauffe, A. Casulli, and A. Massolo. 2025. New evidence from the northern Apennines, Italy, suggests a southward expansion of Echinococcus multilocularis range in Europe. Scientific reports 15:7353.
19. Cartuyvels, E., T. Adriaens, K. Baert, F. Huysentruyt, and K. Van Den Berge. 2022. Prevalence of fox tapeworm in invasive muskrats in Flanders (north Belgium). Animals: an open access journal from MDPI 12:879.
20. Casulli, A., Z. Széll, E. Pozio, and T. Sréter. 2010. Spatial distribution and genetic diversity of Echinococcus multilocularis in Hungary. Veterinary parasitology 174:241–246.
21. Cenni, L., A. Simoncini, L. Massetti, A. Rizzoli, H. C. Hauffe, and A. Massolo. 2023. Current and future distribution of a parasite with complex life cycle under global change scenarios: Echinococcus multilocularis in Europe. Global change biology 29:2436–2449.
22. Combès, B., S. Comte, V. Raton, F. Raoul, F. Boué, G. Umhang, S. Favier, C. Dunoyer, N. Woronoff, and P. Giraudoux. 2012. Westward spread of Echinococcus multilocularis in foxes, France, 2005-2010. Emerging infectious diseases 18:2059–2062.
23. Combès, B., S. Comte, V. Raton, F. Raoul, F. Boué, G. Umhang, S. Favier, C. Dunoyer, N. Woronoff, and P. Giraudoux. 2013. Expansion géographique du parasite Echinococcus multilocularis chez le renard en France 57:16–18.
24. Conlon, C. L., J. P. Brigandi, J. L. Frair, C. Michaud-LeBlanc, K. L. Schuler, M. Lejeune, and C. M. Whipps. 2024. Echinococcus multilocularis in New York wildlife: Distribution and genetic diversity of an emerging pathogen. The journal of parasitology 110:697–708.
25. Conraths, F., and P. Maksimov. 2020. Epidemiology of Echinococcus multilocularis infections: A review of the present knowledge and of the situation in Germany. Berliner und Münchener Tierärztliche Wochenschrift 133.
26. Craig, P. S., L. Deshan, C. N. MacPherson, S. Dazhong, D. Reynolds, G. Barnish, B. Gottstein, and W. Zhirong. 1992. A large focus of alveolar echinococcosis in central China. Lancet 340:826–831.
27. Crotti, S., L. Brustenga, D. Cruciani, P. Bonelli, N. D’Avino, A. Felici, B. Morandi, C. Sebastiani, S. Spina, and M. Gobbi. 2023. Molecular Screening of Echinococcus spp. and Other Cestodes in Wild Carnivores from Central Italy. Veterinary science in China 10.
28. Evason, M. D., A. S. Peregrine, E. J. Jenkins, C. E. Lozoya, L. L. Rund, J. S. Weese, P. D. J. Castro, and C. M. Leutenegger. 2025. Emerging Echinococcus tapeworms: fecal PCR detection of Echinococcus multilocularis in 26 dogs from the United States and Canada (2022-2024). Journal of the American Veterinary Medical Association 263:1–5.
29. Feng, X., X. Qi, L. Yang, X. Duan, B. Fang, Q. Gongsang, B. Bartholomot, D. A. Vuitton, H. Wen, and P. S. Craig. 2015. Human cystic and alveolar echinococcosis in the Tibet Autonomous Region (TAR), China. Journal of helminthology 89:671–679.
30. Gesy, K., J. E. Hill, H. Schwantje, S. Liccioli, and E. J. Jenkins. 2013. Establishment of a European-type strain of Echinococcus multilocularis in Canadian wildlife. Parasitology 140:1133–1137.
31. Giraudoux, P., F. Raoul, E. Afonso, I. Ziadinov, Y. Yang, L. Li, T. Li, J.-P. Quéré, X. Feng, Q. Wang, H. Wen, A. Ito, and P. S. Craig. 2013. Transmission ecosystems of Echinococcus multilocularis in China and Central Asia. Parasitology 140:1655–1666.
32. Gong, Q.-L., G.-Y. Ge, Q. Wang, T. Tian, F. Liu, N.-C. Diao, L.-B. Nie, Y. Zong, J.-M. Li, K. Shi, X. Leng, and R. Du. 2021. Meta-analysis of the prevalence of Echinococcus in dogs in China from 2010 to 2019. PLoS neglected tropical diseases 15:e0009268.
33. Han, J., G. Bao, D. Zhang, P. Gao, T. Wu, P. Craig, P. Giraudoux, X. Chen, Q. Xin, L. He, G. Chen, and T. Jing. 2015. A newly discovered epidemic area of Echinococcus multilocularis in west Gansu Province in China. PloS one 10:e0132731.
34. Heidari, Z., M. Sharbatkhori, I. Mobedi, S. H. Mirhendi, B. Nikmanesh, M. Sharifdini, M. Mohebali, Z. Zarei, K. Arzamani, and E. B. Kia. 2019. Echinococcus multilocularis and Echinococcus granulosus in canines in North-Khorasan Province, northeastern Iran, identified using morphology and genetic characterization of mitochondrial DNA. Parasites & vectors 12:606.
35. Houston, S., S. Belga, K. Buttenschoen, R. Cooper, S. Girgis, B. Gottstein, G. Low, A. Massolo, C. MacDonald, N. Müller, J. Preiksaitis, P. Sarlieve, S. Vaughan, and K. Kowalewska-Grochowska. 2021. Epidemiological and clinical characteristics of alveolar echinococcosis: An emerging infectious disease in Alberta, Canada. The American journal of tropical medicine and hygiene 104:1863–1869.
36. Ito, A., G. Agvaandaram, O.-E. Bat-Ochir, B. Chuluunbaatar, N. Gonchigsenghe, T. Yanagida, Y. Sako, N. Myadagsuren, T. Dorjsuren, K. Nakaya, M. Nakao, Y. Ishikawa, A. Davaajav, and N. Dulmaa. 2010. Histopathological, serological, and molecular confirmation of indigenous alveolar echinococcosis cases in Mongolia. The American journal of tropical medicine and hygiene 82:266–269.
37. Ito, A., and C. M. Budke. 2015. The present situation of echinococcoses in Mongolia. Journal of helminthology 89:680–688.
38. Jeeves, S. P., J. D. Kotwa, B. Stevens, L. Shirose, H. Y. Cai, A. S. Peregrine, S. Mubareka, and C. M. Jardine. 2024. Additional diagnoses of Echinococcus multilocularis in eastern chipmunks (Tamias striatus) from southern Ontario - results from ongoing surveillance for E. multilocularis in intermediate hosts in Ontario, Canada. Veterinary parasitology (Amsterdam: Online) 48:100982.
39. Karamon, J., M. Kochanowski, J. Sroka, T. Cencek, M. Różycki, E. Chmurzyńska, and E. Bilska-Zając. 2014. The prevalence of Echinococcus multilocularis in red foxes in Poland--current results (2009-2013). Parasitology research 113:317–322.
40. Khalid, A., P. K. Muchaal, and D. A. Julien. 2024. Human echinococcosis incidence in Canada: A retrospective descriptive study using administrative hospital and ambulatory visit data, 2000-2020. Releve des maladies transmissibles au Canada [Canada communicable disease report] 50:305–311.
41. Khan, A., G. Umhang, Z. Ullah, F. Boué, V. Bastid, I. Ullah, S. Mahmood, M. S. Afzal, and H. Ahmed. 2021. Investigation of Echinococcus multilocularis in foxes and dogs in Pakistan by detection of copro-DNA. Parasitology research 120:731–737.
42. Kharchenko, V., V. Kornyushin, E. Varodi, and O. Malega. 2008. Occurrence of Echinococcus multilocularis (Cestoda, Taeniidae) in red foxes (Vulpes vulpes) from Western Ukraine. Acta parasitologica 53:36–40.
43. Khuroo, M. S., N. S. Khuroo, and A. A. Rather. 2024. A case series and literature review of Alveolar echinococcosis in Kashmir, India: An emerging endemic zone for Echinococcus multilocularis. Life (Basel, Switzerland) 14:794.
44. Knapp, J., P. Giraudoux, B. Combes, G. Umhang, F. Boué, Z. Said-Ali, S. Aknouche, C. Garcia, M. Vacheyrou, A. Laboissière, V. Raton, S. Comte, S. Favier, J.-M. Demerson, C. Caillot, L. Millon, and F. Raoul. 2018. Rural and urban distribution of wild and domestic carnivore stools in the context of Echinococcus multilocularis environmental exposure. International journal for parasitology 48:937–946.
45. Kolapo, T. U., A. Hay, K. M. Gesy, C. F. Frey, J. L. Rothenburger, D. J. Joffe, T. Spotswood, Y. Huang, A. Massolo, A. S. Peregrine, J. E. Hill, and E. J. Jenkins. 2023. Canine alveolar echinococcosis: An emerging and costly introduced problem in North America. Transboundary and emerging diseases 2023:1–10.
46. Konyaev, S. V., T. Yanagida, G. M. Ingovatova, Y. N. Shoikhet, M. Nakao, Y. Sako, A. Y. Bondarev, and A. Ito. 2012. Molecular identification of human echinococcosis in the Altai region of Russia. Parasitology international 61:711–714.
47. Laurimaa, L., K. Süld, E. Moks, H. Valdmann, G. Umhang, J. Knapp, and U. Saarma. 2015. First report of the zoonotic tapeworm Echinococcus multilocularis in raccoon dogs in Estonia, and comparisons with other countries in Europe. Veterinary parasitology 212:200–205.
48. Lavallée-Bourget, È.-M., C. Fernandez-Prada, A. Massé, P. Turgeon, and J. Arsenault. 2024. Prevalence and geographic distribution of Echinococcus genus in wild canids in southern Québec, Canada. PloS one 19:e0306600.
49. Luga, P., A. Gjata, I. Akshija, L. Mino, V. Gjoni, A. Pilaca, M. Zobi, G. E. Martinez, and J. Richter. 2023. What do we know about the epidemiology and the management of human echinococcosis in Albania? Parasitology research 122:1811–1818.
50. Makarikov, A. A., and N. E. Dokuchaev. 2021. Tapeworms in rodents from the Lower Anabar River Basin, with a review of species diversity of cestodes in Yakutia, Russia. Acta parasitologica 66:1012–1020.
51. Malczewski, A., B. Rocki, A. Ramisz, and J. Eckert. 1995. Echinococcus multilocularis (Cestoda), the causative agent of alveolar echinococcosis in humans: first record in Poland. The journal of parasitology 81:318–321.
52. Manfredi, M. T., C. Genchi, R. Deplazes, K. Trevisiol, and C. Fraquelli. 2002. Echinococcus multilocularis infection in red foxes in italy. The veterinary record 150:757.
53. Marcinkutė, A., M. Šarkūnas, E. Moks, U. Saarma, P. Jokelainen, G. Bagrade, S. Laivacuma, K. Strupas, V. Sokolovas, and P. Deplazes. 2015. Echinococcus infections in the Baltic region. Veterinary parasitology 213:121–131.
54. Marinković, D., P. Gavrilović, D. Vidanović, D. Ćirović, M. Kuručki, N. Vasković, and M. Aničić. 2022. First Report of Alveolar Hydatid Disease (Echinococcus multilocularis) in a Golden Jackal (Canis aureus). Acta parasitologica 67:1401–1406.
55. Massolo, A., D. Valli, M. Wassermann, S. Cavallero, S. D’Amelio, A. Meriggi, E. Torretta, M. Serafini, A. Casulli, L. Zambon, C. B. Boni, M. Ori, T. Romig, and F. Macchioni. 2018. Unexpected Echinococcus multilocularis infections in shepherd dogs and wolves in south-western Italian Alps: A new endemic area? International journal for parasitology. Parasites and wildlife 7:309–316.
56. Melotti, J. R., P. M. Muzzall, D. J. O’Brien, T. M. Cooley, and J. I. Tsao. 2015. Low Prevalence ofEchinococcus multilocularisin Michigan, U.S.A.: A Survey of Coyotes (Canis latrans), Red Foxes (Vulpes vulpes), and Gray Foxes (Urocyon cinereoargenteus), 2009–2012. Comparative parasitology 82:285–290.
57. Miller, A. L., G. E. Olsson, M. R. Walburg, S. Sollenberg, M. Skarin, C. Ley, H. Wahlström, and J. Höglund. 2016. First identification of Echinococcus multilocularis in rodent intermediate hosts in Sweden. International journal for parasitology. Parasites and wildlife 5:56–63.
58. Mobedi, I., and A. Sadighian. 1971. Echinococcus multilocularis Leuckart, 1863, in red foxes, Vulpes vulpes Linn., in Moghan, Azerbaijan Province, northwest of Iran. The journal of parasitology 57:493.
59. Moks, E., U. Saarma, and H. Valdmann. 2005. Echinococcus multilocularis in Estonia. Emerging infectious diseases 11:1973–1974.

    Omeragić, J., T. Goletić, A. Softić, Š. Goletić, N. Kapo, D. K. Soldo, J. Šupić, V. Škapur, G. Čerkez, E. Ademović, O. Semren, and A. Alić. 2022. First detection of Echinococcus multilocularis in Bosnia and Herzegovina. International journal for parasitology. Parasites and wildlife 19:269–272.
60. Osterman Lind, E., M. Juremalm, D. Christensson, S. Widgren, G. Hallgren, E. O. Ågren, H. Uhlhorn, A. Lindberg, M. Cedersmyg, and H. Wahlström. 2011. First detection of Echinococcus multilocularis in Sweden, February to March 2011. Euro surveillance : bulletin Europeen sur les maladies transmissibles [Euro surveillance : European communicable disease bulletin] 16:19836.
61. Peregrine, A. S., E. J. Jenkins, B. Barnes, S. Johnson, L. Polley, I. K. Barker, B. De Wolf, and B. Gottstein. 2012. Alveolar hydatid disease (Echinococcus multilocularis) in the liver of a Canadian dog in British Columbia, a newly endemic region. The Canadian veterinary journal. La revue veterinaire canadienne 53:870–874.
62. Perrucci, S., M. Maestrini, F. Coppola, M. Di Marco, A. D. Rosso, M. I. Pacini, P. Zintu, and A. Felicioli. 2023. Gray Wolf (Canis lupus italicus) and Red Fox (Vulpes vulpes) Parasite Survey in Anthropized and Natural Areas of Central Italy. Veterinary sciences 10:108.
63. Petersen, H. H., M. N. S. Al-Sabi, H. L. Enemark, C. M. O. Kapel, J. A. Jørgensen, and M. Chriél. 2018. Echinococcus multilocularis in Denmark 2012-2015: high local prevalence in red foxes. Parasitology research 117:2577–2584.
64. Polish, L. B., E. M. O’Connell, T. F. E. Barth, B. Gottstein, A. Zajac, P. C. Gibson, A. Bah, M. Kirchgessner, M. Estrada, M. A. Seguin, and R. Ramirez-Barrios. 2022. European haplotype of *Echinococcus multilocularis* in the United States. The New England journal of medicine 387:1902–1904.
65. Robbins, W. T., O. Galeuzzi, K. Graham, S. J. Greenwood, M. E. B. Jones, M. Buote, and G. A. Conboy. 2022. Echinococcus multilocularis infection in a red fox (Vulpes vulpes) on Prince Edward Island, Canada. The Canadian veterinary journal. La revue veterinaire canadienne 63:962–966.
66. Šarkūnas, M., R. Bružinskaitė, A. Marcinkutė, K. Strupas, V. Sokolovas, A. Mathis, and P. Deplazes. 2010. Emerging alveolar echinococcosis (AE) in humans and high prevalence of Echinococcus multilocularis in foxes and raccoon dogs in Lithuania. Acta Veterinaria Scandinavica 52:S11.
67. Satoh, M., K. Nakaya, M. Nakao, N. Xiao, H. Yamasaki, Y. Sako, Y. Naitoh, S. Kondo, M. Kobayashi, N. Ohtaishi, and A. Ito. 2005. Short report: Echinococcus multilocularis confirmed on Kunashiri Island, 15 kilometers from the eastern part of Hokkaido, Japan. The American journal of tropical medicine and hygiene 72:284–288.
68. Schurer, J. M., E. Bouchard, A. Bryant, S. Revell, G. Chavis, A. Lichtenwalner, and E. J. Jenkins. 2018. Echinococcus in wild canids in Québec (Canada) and Maine (USA). PLoS neglected tropical diseases 12:e0006712.
69. Schurer, J. M., M. Pawlik, A. Huber, B. Elkin, H. D. Cluff, J. D. Pongracz, K. Gesy, B. Wagner, B. Dixon, H. Merks, M. S. Bal, and E. J. Jenkins. 2016. Intestinal parasites of gray wolves (Canis lupus) in northern and western Canada. Canadian journal of zoology 94:643–650.
70. Schweiger, A., R. W. Ammann, D. Candinas, P.-A. Clavien, J. Eckert, B. Gottstein, N. Halkic, B. Muellhaupt, B. M. Prinz, J. Reichen, P. E. Tarr, P. R. Torgerson, and P. Deplazes. 2007. Human alveolar echinococcosis after fox population increase, Switzerland. Emerging infectious diseases 13:878–882.
71. Shaikenov, B. S. 2006. Distribution and ecology of Echinococcus multilocularis in Central Asia. Parasitology international 55 Suppl:S213-9.
72. Sikó, S. B., P. Deplazes, C. Ceica, C. S. Tivadar, I. Bogolin, S. Popescu, and V. Cozma. 2011. Echinococcus multilocularis in south-eastern Europe (Romania). Parasitology research 108:1093–1097.
73. Stefaniak, M., M. Derda, P. Zmora, and S. P. Nowak. 2023. Risk factors and the character of clinical course of the Echinococcus multilocularis infection in patients in Poland. Pathogens 12:199.
74. Storandt, S. T., and K. R. Kazacos. 1993. Echinococcus multilocularis identified in Indiana, Ohio, and east-central Illinois. The Journal of parasitology 79:301–305.
75. Storandt, S. T., and K. R. Kazacos. 2012. Echinococcus multilocularis identified in Michigan with additional records from Ohio. The Journal of parasitology 98:891–893.
76. Storandt, S. T., D. R. Virchow, M. W. Dryden, S. E. Hygnstrom, and K. R. Kazacos. 2002. Distribution and prevalence of Echinococcus multilocularis in wild predators in Nebraska, Kansas, and Wyoming. The Journal of parasitology 88:420–422.
77. Széll, Z., G. Marucci, E. Pozio, and T. Sréter. 2013. Echinococcus multilocularis and Trichinella spiralis in golden jackals (Canis aureus) of Hungary. Veterinary parasitology 197:393–396.
78. Takumi, K., A. de Vries, M. L. Chu, J. Mulder, P. Teunis, and J. van der Giessen. 2008. Evidence for an increasing presence of Echinococcus multilocularis in foxes in The Netherlands. International journal for parasitology 38:571–578.
79. Tang, C. T., Y. C. Quian, Y. M. Kang, G. W. Cui, H. C. Lu, L. M. Shu, Y. H. Wang, and L. Tang. 2004a. Study on the ecological distribution of alveolar Echinococcus in Hulunbeier Pasture of Inner Mongolia, China. Parasitology 128:187–194.
80. Tang, C. T., Y. C. Quian, Y. M. Kang, G. W. Cui, H. C. Lu, L. M. Shu, Y. H. Wang, and L. Tang. 2004b. Study on the ecological distribution of alveolar Echinococcus in Hulunbeier Pasture of Inner Mongolia, China. Parasitology 128:187–194.
81. Tsukada, H., K. Yagi, and Y. Morishima. 2024. Food habits of feral dogs and red foxes in a new endemic area of Echinococcus multilocularis. Mammal study 49:69–75.
82. Umhang, G., J. Knapp, V. Hormaz, F. Raoul, and F. Boué. (n.d.). Proposition d’un scénario spatio-temporel de l’expansion d’E. multilocularis en France grâce à la génétique.
83. Umhang, G., C. Richomme, C. Caillot, V. Bastid, J.-M. Boucher, J.-L. Moyen, C. Novella, B. Richoux, B. Davoust, and F. Boué. 2022. Towards delimitation of the Echinococcus multilocularis parasite’s southernmost range in France. Veterinary parasitology (Amsterdam: Online) 30:100724.
84. Vervaeke, M., P. Dorny, F. Vercammen, S. Geerts, J. Brandt, K. Van Den Berge, and R. Verhagen. 2003. Echinococcus multilocularis (Cestoda, Taeniidae) in Red foxes (Vulpes vulpes) in northern Belgium. Veterinary parasitology 115:257–263.
85. Vervaeke, M., J. van der Giessen, B. Brochier, B. Losson, K. Jordaens, R. Verhagen, C. de L. Coulander, and P. Teunis. 2006. Spatial spreading of Echinococcus multilocularis in Red foxes (Vulpes vulpes) across nation borders in Western Europe. Preventive veterinary medicine 76:137–150.
86. Vuitton, D., Q. Wang, H. Zhou, F. Raoul, J. Knapp, S. Bresson-Hadni, H. Wen, and P. Giraudoux. 2011. A historical view of alveolar echinococcosis, 160 years after the discovery of the first case in humans: part 1. What have we learnt on the distribution of the disease and on its parasitic agent? Chinese medical journal 124:2943–2953.
87. Wahlström, H., H. L. Enemark, R. K. Davidson, and A. Oksanen. 2015. Present status, actions taken and future considerations due to the findings of E. multilocularis in two Scandinavian countries. Veterinary parasitology 213:172–181.
88. Wang, L., G. Quzhen, M. Qin, Z. Liu, H. Pang, R. Frutos, and L. Gavotte. 2022. Geographic distribution and prevalence of human echinococcosis at the township level in the Tibet Autonomous Region. Infectious diseases of poverty 11:10.
89. Wang, L.-Y., M. Qin, Z.-H. Liu, W.-P. Wu, N. Xiao, X.-N. Zhou, S. Manguin, L. Gavotte, and R. Frutos. 2021. Prevalence and spatial distribution characteristics of human echinococcosis in China. PLoS neglected tropical diseases 15:e0009996.
90. Williams, L. B. A., and N. Walzthoni. 2023. Diagnosis, treatment, and outcome of four dogs with alveolar echinococcosis in the northwestern United States. Journal of the American Veterinary Medical Association 261:1–6.
91. Zajac, A., D. Fairman, E. McGee, B. Wells, A. Peregrine, E. Jenkins, T. LeRoith, and B. St John. 2020. Alveolar echinococcosis in a dog in the eastern United States. Journal of veterinary diagnostic investigation: official publication of the American Association of Veterinary Laboratory Diagnosticians, Inc 32:742–746.
92. Ziadinov, I., P. Deplazes, A. Mathis, B. Mutunova, K. Abdykerimov, R. Nurgaziev, and P. R. Torgerson. 2010. Frequency distribution of Echinococcus multilocularis and other helminths of foxes in Kyrgyzstan. Veterinary parasitology 171:286–292.
