# Supplementary material for: Detection of Echinococcus multilocularis in coyotes in Washington State, USA highlights need for increased wildlife surveillance
Source: PLoS Negl Trop Dis. 2026 Mar 24;20(3):e0013502. doi: 10.1371/journal.pntd.0013502 (PMC13012483; doi:10.1371/journal.pntd.0013502)
Supplement: S2 Text — A text description of the protocol is available in the Methods section. (DOCX) [file pntd.0013502.s010.docx]

Coyote GI tract dissection modified SFCT protocol (with voiceover): <https://youtu.be/FMDNvCuNHCM>
Coyote GI tract dissection modified SFCT timelapse:

https://youtu.be/zrSVC8u3E54
