# Supplementary material for: Detection of Echinococcus multilocularis in coyotes in Washington State, USA highlights need for increased wildlife surveillance
Source: PLoS Negl Trop Dis. 2026 Mar 24;20(3):e0013502. doi: 10.1371/journal.pntd.0013502 (PMC13012483; doi:10.1371/journal.pntd.0013502)
Supplement: Attachment — Submitted filename: Review of PNTD.pdf [file pntd.0013502.s011.pdf]

Review of PNTD-D-25-01487

"Detection of *Echinococcus multilocularis* in coyotes in Washington State, USA highlights need for increased global wildlife surveillance"

This paper is suitable for publication in PNTD, and Echinococcosis is a listed group within the 21 NTDs. This study reports an important finding – namely new reports of *Echinococcus multilocularis*, a serious zoonosis, in reservoir hosts in a newly endemic region of the USA. Concerningly, many positives were in highly populated regions, posing a risk for both canine and human health, and prevalence (37%) is high. The search for this parasite in coyotes coincided with detection of this parasite in dogs (as both definitive and intermediate hosts) as an early warning signal. The methods are well described and the combination of morphology and molecular identification are compelling. The authors also developed a novel metabarcoding approach to differentiate among different canid cestodes, which is quite exciting and promising for wildlife surveillance, where mixed taeniid infections are the rule, rendering Sanger sequencing ineffective.

There is one important study limitation: the method used to extract DNA from field collected feces (freeze thaw cycles and a commercial extraction kit) is not the recommended procedure for extracting DNA from tough taeniid eggs, which ideally uses a bead based mechanical disruption step. As well, the authors do not describe how much fecal matter was used for DNA extraction – if only a swab, this is a tiny amount and therefore quite likely to miss low egg intensities and non-uniform distribution within feces. This methodological limitation most likely accounts for the lack of sensitivity of the fecal based sampling, and the authors should note this study limitation and be cautious when advocating against non-invasive studies, which are increasingly used, and useful.

This paper very thoroughly reviews the literature, which is commendable, but since this is a primary data submission vs a review, it should be substantially shortened and the focus sharpened on the North American situation. Alveolar echinococcosis is a well known NTD and the amount of background given in the introduction could be reduced accordingly.

Authors need to be a bit careful in the conclusions: for example: "this is the first confirmation of *E. multilocularis* in a wild host on the west coast of the U.S." - the parasite has been well described in wild canids (arctic and red fox) on the west coast of Alaska.

Overall, a well written and enjoyable read, which can be pruned to focus more clearly on this study and its significance within the broader context of an emerging and serious threat to canine and public health in North America.

Detailed comments:

Title: While I firmly agree that global wildlife surveillance is much to be desired for *Echinococcus* spp., some might argue that this cannot be concluded from the results of this study.

Introduction:

Line 62: "Historically considered a boreal- and tundra-region parasite"- the parasite has long been established in the prairie regions of central North America, and indeed recent findings in boreal regions in Canada was a bit surprising.

Line 87:"Pathologically significant infections have been reported in coyotes, gray wolves" This is confusing – the intestinal form of *E. multilocularis* is generally associated with minimal pathology in canid definitive hosts?

Line 112:"However, a rapid expansion of *E. multilocularis* in wild canid hosts across North America has occurred throughout the past decade, starting in the Canadian province of Alberta (Figure 2; [10–18])."

While Alberta is currently the hotspot of *E. multilocularis*, this somewhat reflects sampling effort, and it should be noted that European type *E. multilocularis* was first detected in a dog and coyotes in central British Columbia.

Line 127"Canadian provinces and U.S. states that have reported *E. multilocularis* attributed to "Asian and European strains": Asian strains have only been described in western Alaska within North America, to the best of this reviewer's knowledge (see Nakao et al., 2009).

Line 145: I would not call reports of *E. multilocularis* in feces or intestines of dogs as anomalous, since they are natural definitive hosts. Unless the author is specifically referring to dogs as aberrant intermediate hosts?

Methods:

Study area could be described more succinctly, unless habitat type, climate, and human density were variables analyzed in relation to prevalence. Likewise, a single sentence justifying the choice of coyotes for sampling would likely suffice.

Line 198-99: Intermediate hosts in North America do not include squirrels, rats, or beavers. The Deplazes review cited here notes deer mice, meadow voles, house mice, bushy tailed wood rat, and lemmings, voles and shrews in Alaska. More recent studies include chipmunks and muskrats.

Line 206-207 – briefly describe PPE and precautions to prevent human exposure during recovery of intestines at necropsy.

Line 212 and elsewhere: please make it clear whether you are targeting host DNA (to confirm individual coyote identification), or DNA for dietary analysis, or DNA for cestodes. Likewise, metabarcoding for dietary vs cestode identification.

Lines 219-232: if using a published method, only describe modifications from that method

Line 238: I generally prefer helminth or cestode to "worm", but author's choice.

Lines 244-253 could be concisely stated as " adult cestodes of *E. multilocularis* were differentiated from those of *E. granulosus* based on total length of the adult worm, the ratio of the length of the gravid proglottids to the total length, the location of the genital pore within each proglottid, and the shape of the uterus (57,58)"

Line 253: was a flotation performed to see if taeniid eggs were present in feces? This is a very common method used in dogs and wild canids, and the comparison would have been interesting.

Line 257 and throughout: clearly state when targeting DNA or metabarcoding for cestodes vs coyote identification vs dietary, as this is confusing. A criticism of ground collected samples is that the canid species cannot always be determined (i.e. dog vs coyote) – were all environmental samples confirmed as coyote? It is commendable that the study was able to identify coyotes to individual level. This is a study strength.

Line 262: how much fecal matter was used for DNA extraction? Only a swab? That is a very small amount. Freeze thaw cycles are not effective to crack taeniid eggs and mechanical disruption methods (bead bashing) using larger amounts of feces (vs swabs) should be considered the gold standard.

Line 284: I am not currently able to see S1, but it will be important to demonstrate that these primers amplified and differentiated the most common differential diagnoses for taeniid eggs in coyotes, such as *E. canadensis, E. granulosus sensu lato, E. multilocularis, Taenia pisiformis, Taenia hydatigena, Taenia krabbei, Taenia polyacantha* etc…

Line 316: check spelling of Nanak. Why run as 5 singleplex PCRs?

Table 2: It is interesting that prevalence was higher in the carcasses. Does this reflect the poor sensitivity of the DNA extraction method from scat, or are the animals that are found dead more likely to be infected (younger, more likely to eat rodents vs larger prey or garbage?)

Discussion: general comment – focus on the findings of this study, and discuss only the literature needed to place these findings into context.

Line 438: why 2021? Ie why not earlier?

Line 438: why mtG h28 vs the Genbank accession number?

Line 443: the BC1 and other E3/E4 haplotypes have been reported in SK coyotes as well – see reference 30. Almost all isolates from dogs and wild canids in western North America have been E3/E4 haplotypes, summarized in https://doi.org/10.1016/bs.apar.2025.07.003. This reinforces the supposition that this has likely been circulating for some time below the radar.

Line 466-479: It is important to note that the methods used here, and in many commercial kits, would not be sufficient to crack taeniid eggs and release DNA from eggs so it can be extracted and purified. So please do not give up on DNA based methods for screening ground collected feces. Many European countries use non invasive fecal surveillance to good effect on fox scat (without recovering eggs first, which is tedious). However, it is a valid point that adapting methods suitable for dietary analyses to parasite screening may require significantly increasing the amount of scat analyzed (more than a swab, 1 g or more) and must include an additional, proven effective method for egg cracking.

Line 488: *E multilocularis* likely has more freeze resistance, since it is present above 70 degrees of latitude, while *T. pisiformis* is more of a subarctic parasite. So it might be that freeze thaw more effectively released DNA from eggs of *T. pisiformis* than *E. multilocularis*. Interesting ideas for future research!

Lines 512-514: most European countries have moved away from SFCT in favor of fecal or intestinal PCR, for all the reasons that you list ☺ The real promise of this novel metabarcoding method on intestinal or fecal samples is managing coinfections, which are common in wildlife and make Sanger sequencing difficult.

Line 522: Prevalence in coyotes in Saskatchewan was about 70% (reference 68), and is around 51% in AB and SK overall (https://doi.org/10.1016/bs.apar.2025.07.003). So perhaps this finding is comparable to highly endemic regions -still quite concerning!

Line 552-573: can be significantly condensed, focusing on the dietary findings and the known intermediate hosts in North America to suggest the most likely rodent candidates in which to go looking.

Lines 575-612: can be significantly condensed, as much is beyond the scope of the data of the current study. A brief mention of concerns for dogs and humans is appropriate, and the role of veterinarians, public health, and regulatory measures for control. Recommendations for control should be based on cited literature and not opinion (as much as I agree with the author's opinions!).

Figure 1 should be removed. It is beyond the scope of the paper and is not relevant to the North American situation. It is not clear what methods were used to find peer reviewed papers, nor value added beyond the Deplazes et al 2017 review.

Figure 2 misses reports of *E. multilocularis* in wolves in the Northwest Territories (doi:10.1017/S0031182013001716). Please describe in a bit more detail the methods used to

find peer reviewed studies – databases, search terms etc – and if this includes reports in any host, including people or dogs with travel history.

Figure 3: A lovely life cycle. The fecal matter is a bit confusing as it looks rather worm like, and the expectation was to see eggs? And the depiction of the metacestode "cyst" of alveolar echinococcosis is also a bit nonstandard, looking rather egg like.
