# Supplementary material for: Detection of Echinococcus multilocularis in coyotes in Washington State, USA highlights need for increased wildlife surveillance
Source: PLoS Negl Trop Dis. 2026 Mar 24;20(3):e0013502. doi: 10.1371/journal.pntd.0013502 (PMC13012483; doi:10.1371/journal.pntd.0013502)
Supplement: Attachment — Submitted filename: Response to Reviewers.pdf [file pntd.0013502.s013.pdf]

Reviewer 1

General comments

This is a very important and relevant study on the expansion and characterization of Echinococcus multilocularis in a novel geographic range in North America. The authors conducted a robust surveillance study and discovered a high prevalence of E. multilocularis in wildlife in a region lacking previous surveillance efforts. This is highly significant as this parasite can cause a fatal disease in humans, and is currently underreported and misdiagnosed, even in endemic areas. Continued surveillance, reporting, and education is vital to understanding and controlling this parasite, especially as emerges in novel areas within the United States.

This study is well-written, with very well-done figures, and detailed conclusions on the work. A few structural changes should be made to the introduction to keep it focused and organized to E. multilocularis knowledge in North America, where the study takes place. More information is needed on the molecular methodology and results, and accession numbers for the genes used to identify the haplotypes detected in this study should be included, so that they can be openly compared to the haplotypes being detected across North America. Specific comments and questions addressed in the sections above. I recommend that this publication be accepted with minor revisions.

Thank you for your thoughtful and encouraging assessment of our manuscript. We agree that the introduction can be significantly condensed to remain in alignment with the study focus, and have reorganized this section accordingly. We also agree that the GenBank accession numbers should be added in order to simplify comparisons — we had significant difficulty tracking down haplotype sequences across the literature and wish to facilitate future work. Specific comments are addressed individually below.

Methods

Section starting on 310: Why was only one worm from each positive carcass used for haplotype identification? Did you check the amplified DNA markers for overlapping peaks that could represent mixed infections? Homogenizing pooled samples of a subset of worms from a carcass and sequencing that DNA could reveal if there were multiple haplotypes present in an infected individual.

Sanger sequencing was used for *E. multilocularis* haplotype determination only on single worms due to cost limitations. All sequence chromatograms were checked manually to ensure quality control, and finalized sequences were assembled from forward and reverse reads. The single worms had been rinsed and stored in ethanol prior to DNA extraction, so it was unlikely that we would have detected multiple haplotypes.

While the suggestion to sequence mixed samples would have allowed us to check for overlapping peaks, it would not have allowed us to parse the haplotype sequences from the mixed-signal Sanger sequence. Additionally, mixed signals can have multiple causes and are not always indicative of a mixed-haplotype sample. We plan to conduct additional sequencing in the future, including homogenized pools as suggested by the reviewer. However, additional lab work at this time is cost-

and time-prohibitive, and we feel that the discovery of *E. multilocularis* is the main message of this manuscript.

- Line 388 – what do you mean by 6 coyotes- do you mean 6 worms isolated from 6 different infected coyotes? Why did you use only 6 if you had 37 positive individuals? Why do you think 2 didn't work- were they not infected with E. multilocularis? If the DNA was extracted from worms isolated from infected coyotes, and the PCR didn't work on DNA from these worms- are these worms not E. multilocularis? Were there any other steps taken to identify these DNA/these worms?

Thank you for these questions which will help us to improve the clarity of this section. Yes, we identified 6 worms from 6 different infected coyotes. This manuscript arose from a pilot study to test the method of DNA metabarcoding for detecting helminth communities in coyotes, as well as to develop primers for this purpose. The discovery of *E. multilocularis* was an unexpected surprise. We had been focusing on morphological identification of larger worms from our dissections (i.e., 1-mm sieve contents from the SFCT protocol) up until we received the pilot metabarcoding results, at which point we shifted gears. As a result, our subsequent haplotype analyses were limited to coyote individuals who had confirmed *Echinococcus* morphological or genetic data at that point in time.

As for the 2 that didn't work, worms from these individuals were in poor condition due to being stored in 70% ethanol with a large volume of parasites relative to the volume of ethanol. We believe that DNA was degraded in the specimens we selected.

- Line 388-392: What gene was sequenced? Please include accession numbers for the haplotypes listed in figure 5 and the accession number for the gene you generated. Did you generate sequences from all 3 target genes (nad2, cob, cox1) or just one?

We generated sequences for nad2, cob, and cox1 and have updated the text to clarify this (line 364). We have added the accession numbers for the sequences we generated (line 369-370). GenBank numbers for each haplotype in Figure 5 (now Figure 4) are listed in the comprehensive Table 1 in Lallemand et al. (2024). Instead of creating an identical table, we have added a line to the caption of Figure 4 indicating where readers can find accession numbers: "*GenBank accession numbers for each haplotype are available in Table 1 of Lallemand et al.*"

- Line 392: Figure 5, not 3.

Thank you for catching this error!

Results

Figure 1, 2, and 4 look very good, are of great quality and are accurate to the literature (F1 and F2, F4 is new data). Figure 3 could be moved to the beginning of the paper because it fits the introduction better. Figure 1 is interesting, but as the paper is not focused on defining the global distribution of E. multilocularis, it could be removed.

We appreciate the compliments on these maps and the feedback to improve their placement in the manuscript. We have moved Figure 1 to the supplementary materials; while we agree that it is not

central to the message of the paper, we do not know of other publications that have visualized the chronological spread of *E. multilocularis* in this manner and believe it will be useful for future researchers. We have moved the North America map (formerly Figure 2; now Figure 1) in the place of the Europe/Asia map and have also moved the life cycle figure (formerly Figure 3) to appear after paragraph 2 of the introduction.

- The data in table 2 should be split to represent:
1) Only the prevalence of E. multilocularis in samples tested (carcass and fecal samples, and how many unique individuals were represented in both sample types), giving the 37%, or 37/100 prevalence information clearly.

2) The difference between E. multilocularis identification between the morphological ID, fecal samples from carcass, and intestinal swabs from carcass. This information is confusing in the full table, and it will allow you to break up the table description. This will also provide clearer evidence for the use of different methods and best practices for E. multilocularis detection.

Thank you for this suggestion. Based on feedback from 2 reviewers, we have split Table 2 into 2 tables — one with summarized results similar to that suggested here (new Table 2), and one showing the differences amongst sample types (Table 3).

- Figure 6- Well done staining and picture.

Thank you!

Editorial modifications

Lines 69-72. These two sentences are not linked clearly (spread in Europe canid distribution). Also, lines 70 and 72, "Given the geographically expansive distributions of canid species – the definitive host of E. multilocularis – zoonotic spillover commonly occurs to humans and their pets (19,20)." – this is not accurate, E. multilocularis spillover to humans and pets is quite rare, and has much to do with the prevalence of E. multilocularis in canids versus just the available host distribution. Be clear with the data from the two papers (Davidson et al. 2016 and Crotti et al. 2023) that is being used to support this.

We have removed the sentence on spread in Europe due to feedback from another reviewer. The reviewer is correct in that zoonotic spillover is indeed quite rare. We also thank the reviewer for catching a citation mistake here; we meant to cite source 21 (Oksanen et al. 2016) and not Crotti et al. (2023). We have edited the sentence to read as follows (lines 69-72): *"Given the geographically expansive distribution of canid species – the definitive host of E. multilocularis – and relatively high prevalence of the parasite in canids across the Northern Hemisphere (19, 20), zoonotic spillover to humans and their pets occasionally occurs, though the true distribution of human infections is likely underestimated due to a lack of dedicated surveillance (19)."*

- Paragraph that spans lines 82-95: split up to discuss range in one paragraph and hosts in another.

Thank you for this feedback. Another reviewer suggested that we streamline the introduction. We have therefore removed these first few lines on range and added some of that information to paragraph 1; paragraph 2 is now focused on host species.

- Citation needed line 100

Thanks, we have added a citation here.

- Line 182, Potential definitive hosts: missing Latin name for wolf species in the area

As both gray wolves and coyotes were mentioned in the introduction with Latin names, we did not repeat Latin names here. The Latin name was included for the Cascade red fox, as the subspecies was not previously mentioned.

- Line 235: Gloves

Fixed!

- In the discussion- the citation style changes from numbers to full citations, please keep consistent.

Thank you; we have fixed this issue.

Reviewer 2

This paper is suitable for publication in PNTD, and Echinococcosis is a listed group within the 21 NTDs. This study reports an important finding – namely new reports of *Echinococcus multilocularis*, a serious zoonosis, in reservoir hosts in a newly endemic region of the USA. Concerningly, many positives were in highly populated regions, posing a risk for both canine and human health, and prevalence (37%) is high. The search for this parasite in coyotes coincided with detection of this parasite in dogs (as both definitive and intermediate hosts) as an early warning signal. The methods are well described and the combination of morphology and molecular identification are compelling. The authors also developed a novel metabarcoding approach to differentiate among different canid cestodes, which is quite exciting and promising for wildlife surveillance, where mixed taeniid infections are the rule, rendering Sanger sequencing ineffective.

There is one important study limitation: the method used to extract DNA from field collected feces (freeze thaw cycles and a commercial extraction kit) is not the recommended procedure for extracting DNA from tough taeniid eggs, which ideally uses a bead based mechanical disruption step. As well, the authors do not describe how much fecal matter was used for DNA extraction – if only a swab, this is a tiny amount and therefore quite likely to miss low egg intensities and non-uniform distribution within feces. This methodological limitation most likely accounts for the lack of sensitivity of the fecal based sampling, and the authors should note this study limitation and be cautious when advocating against non-invasive studies, which are increasingly used, and useful.

This paper very thoroughly reviews the literature, which is commendable, but since this is a primary data submission vs a review, it should be substantially shortened and the focus sharpened on the North American situation. Alveolar echinococcosis is a well known NTD and the amount of background given in the introduction could be reduced accordingly.

Authors need to be a bit careful in the conclusions: for example: "this is the first confirmation of *E. multilocularis* in a wild host on the west coast of the U.S." - the parasite has been well described in wild canids (arctic and red fox) on the west coast of Alaska.

Overall, a well written and enjoyable read, which can be pruned to focus more clearly on this study and its significance within the broader context of an emerging and serious threat to canine and public health in North America.

Thank you for your thoughtful and constructive evaluation of our manuscript. We have provided an additional analysis of fecal DNA extraction methods, and we now more cautiously frame the interpretation of non-invasive sampling performance while highlighting the strengths of DNA metabarcoding for low-copy DNA recovery. We have substantially streamlined and refocused the introduction to emphasize the North American context, and we have corrected our wording in the discussion regarding prior detections in Alaska. We have addressed all specific comments below.

Detailed comments:

Title: While I firmly agree that global wildlife surveillance is much to be desired for *Echinococcus* spp., some might argue that this cannot be concluded from the results of this study.

We agree and have removed the word "global" from the title.

Introduction:

Line 62: "Historically considered a boreal- and tundra-region parasite"- the parasite has long been established in the prairie regions of central North America, and indeed recent findings in boreal regions in Canada was a bit surprising.

We agree that this sentence does not fully encompass the historical endemic range of *E. multilocularis*, and is somewhat misleading. We have edited the sentence to read: *"The parasite Echinococcus multilocularis - a cestode of canids that can cause lethal disease in humans - has expanded its range in the past several decades (1,2)."* (lines 62-63)

Line 87: "Pathologically significant infections have been reported in coyotes, gray wolves"- This is confusing – the intestinal form of *E. multilocularis* is generally associated with minimal pathology in canid definitive hosts?

We thank the reviewer for improving the accuracy of this sentence. We have modified the sentence to read "*Definitive host species include coyotes, gray wolves….*" (lines 85-88).

Line 112: "However, a rapid expansion of *E. multilocularis* in wild canid hosts across North America has occurred throughout the past decade, starting in the Canadian province of Alberta (Figure 2; [10–18])." While Alberta is currently the hotspot of *E. multilocularis*, this somewhat reflects sampling effort, and it should be noted that European type *E. multilocularis* was first detected in a dog and coyotes in central British Columbia.

Thank you for correcting this mistake. We have edited the sentence to read *"However, a rapid expansion of E. multilocularis in wild canid hosts across North America has occurred throughout the past decade, most notably in the Canadian province of Alberta, though the first detection was in a dog in central British Columbia (Figure 2; [10–18])"* (lines 112-115). We agree that the impressive sampling effort by researchers in Alberta is part of why it is considered a hotspot, though we did not think discussion of this consideration was within the scope of our introduction.

Line 127: "Canadian provinces and U.S. states that have reported *E. multilocularis* attributed to "Asian and European strains": Asian strains have only been described in western Alaska within North America, to the best of this reviewer's knowledge (see Nakao et al., 2009).

Thank you for this clarification. We have removed the mention of Asian haplotypes here.

Line 145: I would not call reports of *E. multilocularis* in feces or intestines of dogs as anomalous, since they are natural definitive hosts. Unless the author is specifically referring to dogs as aberrant intermediate hosts?

We agree that "anomalous" is an imprecise word choice here. We have moved the word "isolated" here from earlier in the sentence. It now reads: *"Recent reports of E. multilocularis in*

***dogs in the northwestern United States suggest that its distribution is much larger than previously known, but until now, these isolated detections were not linked to an established sylvatic transmission cycle."* (lines 139-141)**

Methods:

Study area could be described more succinctly, unless habitat type, climate, and human density were variables analyzed in relation to prevalence. Likewise, a single sentence justifying the choice of coyotes for sampling would likely suffice.

We agree that this section can be significantly streamlined and have shortened it accordingly. We have moved the text in "Potential intermediate hosts" to the discussion as suggested by another reviewer. However, we chose to keep the information on other definitive hosts, as both gray wolves and Cascade red foxes are both heavily studied species in our region.

Line 198-99: Intermediate hosts in North America do not include squirrels, rats, or beavers. The Deplazes review cited here notes deer mice, meadow voles, house mice, bushy tailed wood rat, and lemmings, voles and shrews in Alaska. More recent studies include chipmunks and muskrats.

Thank you for pointing out this oversight. We indeed meant that species in these groups (i.e. squirrels, rats, and beavers) have been implicated as intermediate hosts in Europe and Asia (as reviewed in citation 26, Romig & Wasserman 2024), and may therefore also be potential intermediate hosts in North America. However, we recognize that this is confusing and beyond scope, and have edited the sentence as such (lines 545-548): *"Cricetid rodents are the most common intermediate hosts in North America, with deer mice (Peromyscus spp.) and voles (Microtus spp.) thought to be responsible for the vast majority of transmission (22), but other rodents such as muskrats (Odantra zibethicus) and chipmunks (family Sciuridae) have also been implicated (26)."*

Line 206-207 – briefly describe PPE and precautions to prevent human exposure during recovery of intestines at necropsy.

Thanks for this important comment. We have added details on these precautions (lines 176-180): *"Gross necropsies were performed at the University of Washington Burke Museum of Natural History and Culture biology specimen preparation laboratory, a Biosafety Level 2 laboratory. Lab coats and nitrile gloves were worn during necropsies. Gastrointestinal tracts were tied off at the stomach and rectum, carefully removed with gloved hands, placed into zip-top bags, and saved for further dissection."*

Line 212 and elsewhere: please make it clear whether you are targeting host DNA (to confirm individual coyote identification), or DNA for dietary analysis, or DNA for cestodes. Likewise, metabarcoding for dietary vs cestode identification.

We have clarified throughout in the "Carcass dissection and worm collection" sections.

Lines 219-232: if using a published method, only describe modifications from that method

We appreciate the reviewer's suggestion to streamline the methods. Our method is close to but modified slightly from Gesy et al. (2013), and we believe these changes are important enough to warrant description, as they may be of help to future researchers. Particularly, the method of stacking the two sieves on top of each other increases efficiency of the dissection and was devised by an author on this manuscript. Additionally, Gesy et al. did not include protocols for molecular sampling (i.e., swabbing, fecal sample collection), and we thought it important to describe at which point we collected these samples. We have included a Youtube video of our dissection protocol to help future researchers (Supporting Information S10).

Line 238: I generally prefer helminth or cestode to "worm", but author's choice.

Agreed, and changed to "Helminths were identified…" (line 220).

Lines 244-253 could be concisely stated as " adult cestodes of *E. multilocularis* were differentiated from those of *E. granulosus* based on total length of the adult worm, the ratio of the length of the gravid proglottids to the total length, the location of the genital pore within each proglottid, and the shape of the uterus (57,58)"

We appreciate this excellent suggestion to shorten this section and have replaced these lines with the sentence suggested by the reviewer (lines 221-224).

Line 253: was a flotation performed to see if taeniid eggs were present in feces? This is a very common method used in dogs and wild canids, and the comparison would have been interesting.

We did not perform fecal floats or sedimentation due to logistic constraints, and instead focused on counting adult specimens from the carcass samples to verify the sensitivity of our primers. However, we agree that comparisons from fecal sedimentation of field-collected feces would have been interesting and plan to conduct this comparison in the future as part of an undergraduate capstone thesis.

Line 257 and throughout: clearly state when targeting DNA or metabarcoding for cestodes vs coyote identification vs dietary, as this is confusing. A criticism of ground collected samples is that the canid species cannot always be determined (i.e. dog vs coyote) – were all environmental samples confirmed as coyote? It is commendable that the study was able to identify coyotes to individual level. This is a study strength.

We have clarified the methods throughout the molecular identification section. All fecal samples were genetically confirmed as coyote. We thank the reviewer for the compliment; we agree that individual identification, while time-consuming, greatly improves the usefulness of field-collected fecal samples.

Line 262: how much fecal matter was used for DNA extraction? Only a swab? That is a very small amount. Freeze thaw cycles are not effective to crack taeniid eggs and mechanical disruption methods (bead bashing) using larger amounts of feces (vs swabs) should be considered the gold standard.

We used 200 mg of fecal matter for the fecal samples collected directly from carcasses. The field-collected feces had already had their DNA extracted as part of a separate diet study, with the extractions being done on swabs that had been rubbed across their outer and inner surfaces (see Kreling 2025). Due to the time and cost limitations that came with the unexpected discovery of *E. multilocularis* in our study area, and because we wanted to test the efficacy of our new worm ID NGS panel, we chose to test our panel on the existing scat swab extractions rather than attempting to re-extract the field scats.

We were also curious about the difference between fecal matter and swabs, especially for fecal samples, where DNA from adult helminths is not abundant. So we conducted an additional analysis directly comparing DNA recovery from fecal matter versus swabs of fecal matter and have included this post-hoc analysis in S9. Our analysis found that swabs of fecal samples indeed performed as well as (or better than) fecal matter samples when it came to helminth detection. We have added lines 242-246 to our "DNA Extraction" section referring to this supplementary table: *"To determine the sensitivity of swabs compared to fecal matter, we conducted an additional analysis comparing sequence read recovery of intestinal swabs, fecal matter, and fecal swabs of randomly selected carcasses (Supporting Information S9). Fecal swabs were equally as sensitive as fecal matter for the closely related taeniid T. pisiformis, and slightly more sensitive than fecal matter for E. multilocularis (Supporting Information S9)."*

The results of our posthoc analysis indicate that next-generation sequencing methods targeting eDNA with low-copy or degraded DNA may detect taeniid DNA in fecal swabs with equal or higher sensitivity as fecal matter samples.

We agree that bead bashing would have been ideal and would have likely increased our sequence read recovery across the 2 fecal sample types, but we were regrettably logistically constrained for this pilot sequencing run. We therefore followed Klein et al. 2014 (DOI 10.1007/s00436-014-4055-x), who described successful recovery of *E. multilocularis* DNA from fecal samples using freeze-thaw cycles.

Line 284: I am not currently able to see S1, but it will be important to demonstrate that these primers amplified and differentiated the most common differential diagnoses for taeniid eggs in coyotes, such as *E. canadensis, E. granulosus sensu lato, E. multilocularis, Taenia pisiformis, Taenia hydatigena, Taenia krabbei, Taenia polyacantha* etc…

We apologize for the technical issues with the supporting information and appreciate the

reviewer's thoroughness here. S1 (now S2) is a table describing the GenBank sequences and positive control samples we used in primer development. Using our positive control samples, we confirmed that primers amplified DNA from *Taenia taeniaeformis*, *Taenia hydatigena*, *Taenia pisiformis*, *Echinococcus multilocularis*, and *Echinococcus granulosus sensu stricto*, and *E. canadensis sensu lato*. Using both sequences from our positive control samples and additional sequences from Genbank, we confirmed that our primers differentiate between all of these species as well as *Taenia hydatigena*, *Taenia krabbei*, *Taenia polyacantha*, and several other taeniid and other cestode species (see S2). We have also added a .fasta file of these alignments (S3) for the reader's convenience.

Line 316: check spelling of Nanak. Why run as 5 singleplex PCRs?

We have fixed the spelling; thanks for catching this. Singleplex PCRs were used in Sanger sequencing methods (as it is impossible to multiplex for Sanger sequencing), but we multiplexed PCRs for NGS analyses.

Table 2: It is interesting that prevalence was higher in the carcasses. Does this reflect the poor sensitivity of the DNA extraction method from scat, or are the animals that are found dead more likely to be infected (younger, more likely to eat rodents vs larger prey or garbage?)

This is a good point. Preliminary data from a forthcoming manuscript titled "Diet and human population density mediate parasite infection burden in coyotes" (in prep; expected submission early 2026) from some of our authors suggests that diet and age group do not impact infection with *E. multilocularis* in our study area. As we successfully recovered sequences of *T. pisiformis* in scat samples, but did not recover *E. multilocularis*, we at first thought that swabs may have been less sensitive for *E. multilocularis* ova specifically. However, our post-hoc analysis (see above) determined this might not be the case. We conclude that this discrepancy is likely due to a combination of lower *E. multilocularis* prevalence in the urban core of the study region (where most of our field-collected scat samples were collected), and higher resistance of *E. multilocularis* eggs to freeze-thaw cycles.

Discussion: general comment – focus on the findings of this study, and discuss only the literature needed to place these findings into context.

Line 438: why 2021? Ie why not earlier?

We began our scat and carcass collection in 2021, so that is when the earliest known infected sample was collected. We have changed the sentence to reflect this (and added the years to the methods). It is likely that coyotes in the region have been infected for much longer, but we cannot know with certainty.

Line 438: why mtG h28 vs the Genbank accession number?

Simply because these names were used by Lallemand et al. (2024), which provides a detailed table with each haplotype name and GenBank accession numbers. Additionally, in some cases, many GenBank sequences fit the same haplotype. We have added clarification in the caption of Figure 4 that multiple GenBank sequences are available for each haplotype in Table 1 of Lallemand et al. (2024).

Line 443: the BC1 and other E3/E4 haplotypes have been reported in SK coyotes as well – see reference 30. Almost all isolates from dogs and wild canids in western North America have been E3/E4 haplotypes, summarized in https://doi.org/10.1016/bs.apar.2025.07.003. This reinforces the supposition that this has likely been circulating for some time below the radar.

Thanks for this clarification. This excellent and much-needed review chapter was published just 2 weeks after we submitted our manuscript! We have changed "British Columbia" to "Canada" and citations to this chapter in lines (423-424): "*To the best of our knowledge, this haplotype has not yet been reported in wildlife outside of Canada (30, 115).*"

Line 466-479: It is important to note that the methods used here, and in many commercial kits, would not be sufficient to crack taeniid eggs and release DNA from eggs so it can be extracted and purified. So please do not give up on DNA based methods for screening ground collected feces. Many European countries use non invasive fecal surveillance to good effect on fox scat (without recovering eggs first, which is tedious). However, it is a valid point that adapting methods suitable for dietary analyses to parasite screening may require significantly increasing the amount of scat analyzed (more than a swab, 1 g or more) and must include an additional, proven effective method for egg cracking.

We agree that mechanical disruption methods such as bead beating would likely improve recovery, and have noted this in lines 460-462: *"However, we did not use additional mechanical disruption steps, such as bead-beating, which probably limited DNA recovery from our samples (61)."* However, based on our posthoc analysis and other manuscripts (see McCosker et al. 2020 https://doi.org/10.1656/045.027.0409, Thomas et al. 2021 https://doi.org/10.1017/S0031182021001840, Couch et al. 2022 https://doi.org/10.1098/rspb.2022.0079, Briscoe et al. 2021 10.1111/1755-0998.13548 – all using kits with 200-250 mg samples), perhaps not as much fecal matter or other eDNA medium is needed when using DNA metabarcoding or other highly sensitive high-throughput sequencing methods specializing in targeting low-copy, trace, or degraded DNA (compared to other methods such as qPCR or Sanger sequencing). There are few papers applying this method for cestodes, let alone taeniids specifically; this would be an interesting area of future research.

Line 488: *E multilocularis* likely has more freeze resistance, since it is present above 70 degrees of latitude, while *T. pisiformis* is more of a subarctic parasite. So it might be that freeze thaw more effectively released DNA from eggs of *T. pisiformis* than *E. multilocularis*. Interesting ideas for future research!

Interesting point! We have added a sentence to this effect: *"E. multilocularis is present as far north as the Arctic region, while Taenia pisiformis (the other taeniid species we detected) is a subarctic parasite, which might explain why E. multilocularis eggs are more freeze-resistant."* (lines 474-476)

Lines 512-514: most European countries have moved away from SFCT in favor of fecal or intestinal PCR, for all the reasons that you list ☺ The real promise of this novel metabarcoding method on intestinal or fecal samples is managing coinfections, which are common in wildlife and make Sanger sequencing difficult.

Thank you for pointing out that most European countries have moved away from SFCT; clearly, we are still a bit behind here in the States :) We have added a sentence clarifying the strengths of metabarcoding (lines 503-505): "*Indeed, the primary strength of DNA metabarcoding is the ability to identify coinfections of multiple helminth species, which are common in wildlife and reduce the efficacy of methods such as Sanger sequencing.*"

Line 522: Prevalence in coyotes in Saskatchewan was about 70% (reference 68), and is around 51% in AB and SK overall (https://doi.org/10.1016/bs.apar.2025.07.003). So perhaps this finding is comparable to highly endemic regions -still quite concerning!

Thanks, this new review is again a very helpful summation of the current situation in North America! We have edited this line accordingly and added citation #68 and the Jenkins et al. review; it now reads "*Our results suggest a much higher prevalence rate than most other similar studies, with the exception of coyote populations in highly endemic regions of the provinces of Saskatchewan and Alberta, Canada (68,89,90,115).*" (lines 512-514)

Line 552-573: can be significantly condensed, focusing on the dietary findings and the known intermediate hosts in North America to suggest the most likely rodent candidates in which to go looking.

We agree and have shortened this section accordingly, though we have also moved coyote dietary details from Methods here based on another reviewer's feedback (paragraph at line 542).

Lines 575-612: can be significantly condensed, as much is beyond the scope of the data of the current study. A brief mention of concerns for dogs and humans is appropriate, and the role of veterinarians, public health, and regulatory measures for control. Recommendations for control should be based on cited literature and not opinion (as much as I agree with the author's opinions!).

We appreciate this comment and have condensed the recommendations and added citations (lines 569-577).

Figure 1 should be removed. It is beyond the scope of the paper and is not relevant to the North American situation. It is not clear what methods were used to find peer reviewed papers, nor value added beyond the Deplazes et al 2017 review.

We have moved Figure 1 to the supplemental information (Figure S1). A description is available

in S8 for the methods used in the former Figure 1 (now figure S1) and the new Figure 1 (formerly Figure 2; North American map). While Deplazes et al. provides a very useful map, their map did not provide a visual representation of the chronological spread of *E. multilocularis*. Additionally, *E. multilocularis* has been detected in multiple new regions since Deplazes et al. was published (e.g., northern Italy, Bosnia and Herzegovina, northern Pakistan). While we agree that Figure 1 does not belong in the main text, we believe it adds additional context and new information not compiled in any map this decade that may be useful to future researchers of *E. multilocularis*.

Figure 2 misses reports of *E. multilocularis* in wolves in the Northwest Territories (doi:10.1017/S0031182013001716). Please describe in a bit more detail the methods used to

find peer reviewed studies – databases, search terms etc – and if this includes reports in any host, including people or dogs with travel history.

Thank you; we apologize for this oversight and have edited Figure 2 (now Figure 1) to include these reports. We have described the methods used to find peer-reviewed studies, and our parameters, in S8. We included reports from all host species (i.e. people, dogs, or wild canids), but only where the authors determined the infection was acquired locally or there was no known travel history.

Figure 3: A lovely life cycle. The fecal matter is a bit confusing as it looks rather worm like, and the expectation was to see eggs? And the depiction of the metacestode "cyst" of alveolar echinococcosis is also a bit nonstandard, looking rather egg like.

Thank you! We have adjusted the figure so the cysts are more "cyst-shaped." The figure shows eggs on the inner cycle next to the coyote feces drawing, which was drawn based on a photograph of a coyote fecal sample.

Reviewer 3

This nicely written manuscript provides new data on the presence and spread of *E. multilocularis* in coyotes in Washington State (USA) and highlights the implications for alveolar echinococcosis risk in the Pacific Northwest.

Gastrointestinal tracts obtained from coyote carcasses, as well as field-collected scats were examined for *Echinococcus* worms and eggs. DNA was extracted from faecal samples, intestinal swabs, and tapeworm material for molecular analysis and *E. multilocularis* haplotype identification. Individual coyotes were identified using microsatellite markers. As a byproduct, the sensitivity of morphological and molecular detection methods was evaluated and compared.

In total, almost half (45.7%) of the 81 examined coyote carcasses were infected with *E. multilocularis*. The *E. multilocularis* haplotype identified in four individual coyotes was previously reported from British Columbia (Canada) and is closely related to haplotypes from France. None of the 30 field-collected scat samples tested positive for *E. multilocularis*, which might be due to a methodical error. This is briefly discussed.

This manuscript is a proper piece of work which confirms that *E. multilocularis* has an established sylvatic life cycle in Washington State. I have only few suggestions for minor amendments and corrections:

Thank you for your positive assessment of our manuscript. We appreciate your constructive suggestions for improving clarity, accuracy, and presentation of our results, which have strengthened the manuscript, and we address each specific point in detail below.

Introduction:

Figure 1: The light-yellow colouration of the endemic areas pre 1990s is barely distinguishable from the white background. Please choose a darker tint for better visibility. This is also the case for Figure 2.

Thanks for pointing this out; we have darkened the map colors accordingly. Figure 1 can now be found in S1.

Line 70: Missing parenthesis – "(Figure 1; (10-18))".

Thank you for catching this error; it's now fixed!

Lines 120-122: It might be interesting to deliberate (either here or in the discussion) if the increase of human AE cases in North America can also be linked to an increased awareness of this disease among health professionals (fewer misdiagnoses).

This is probably an important contribution! We have added a sentence in the discussion: *"Indeed, the recent increase of human AE cases in North America may be due in part to increased awareness of the disease among health professionals (115)."* (lines 592-593)

Methods:

Lines 182-200: The segments 'Potential definitive hosts' and 'Potential intermediate hosts' do not belong in the 'Methods' chapter. As results of a previous study, information on the diet of Washington coyotes may be included as part of the introduction (possible wildlife hosts in Washington) or in the discussion ('Transmission dynamics…').

Thank you for this feedback. We have moved the information in "Potential intermediate hosts" to the suggested section in the discussion, which will improve the flow of the paper (lines 542-551). As we thought it important to contextualize the study area prior to providing rationale for the host species, we have folded the sentences from "Potential definitive hosts" into the previous paragraph, which now summarizes the study area and canid hosts in the region.

Line 193: Period after "(50)" should be a comma.

Thank you - we have replaced the period with a comma.

Lines 201-207: In which years were the carcasses collected? Please add this information.

Carcasses were collected between 2021 and 2024. We have added this information in the first sentence of the section (lines line 175.)

Line 235: The G in "Gloves" is missing.

Thanks; we have added the G in "Gloves."

Lines 272-274: The "freshness categories" should be mentioned in the segment 'Coyote scat collection' rather than here.

We agree and have moved this sentence to the "Coyote scat collection" section, immediately following the first sentence.

Lines 310-315: Why was the haplotype analysis limited to only six positive coyotes? Is there a reason why it was it necessary to confirm *E. multilocularis* both morphologically and with Illumina sequencing before Sanger sequencing? Worms were present in 33 individuals – why not genetically identify them with Sanger sequencing?

We appreciate these questions. The haplotype analysis was limited to 6 positive coyotes simply due to time and costs - the discovery of *E. multilocularis* was a surprise result and we did not originally plan to do any Sanger sequencing. Originally, we were conducting a project focused on developing and testing metabarcoding primers for helminths and identifying helminth communities in coyotes. We were not planning on focusing on a single parasite species, but when we unexpectedly found *E. multilocularis* rather than *E. granulosus*, we decided to conduct a brief round of follow-up sequencing. We had been focusing on morphological identification of larger worms from our dissections (i.e., 1mm sieve contents from the SFCT protocol) until we received the metabarcoding results, at which point we shifted gears. We therefore subsampled a handful of coyotes from across

our study region, which was limited to those with infections confirmed either morphologically or with the pilot metabarcoding data at the time we extracted the Sanger sequencing samples. We hope this answers the reviewer's questions.

Results:

The field-collected scat samples were not screened for the presence of taeniid eggs via flotation, correct? It would have been interesting to note if *T. pisiformis* was detected in all taeniid-positive samples or if other taeniid species/ low egg counts were missed by the NGS approach.

We did not screen the field-collected samples for taeniid eggs via flotation or sedimentation due to time constraints and the original goals of the project (see previous answer). We will likely conduct fecal flotations and sedimentation in the future as part of an undergraduate thesis.

Table 2: Separating the scat results into their "freshness categories" in Table 2 seems rather unnecessary, as *E. multilocularis* was not detected in any of them and the freshness aspect is not further discussed (for example regarding successful *Taenia* detection). It might also make more sense to change the table setup to something like the following, for clarity:

		Available sample type		
Coyote carcass samples	total	Morphological ID	Faecal samples	Intestinal swabs
n dissected individuals	81	76	32	22
E.m. positive	37 (45.7%)	33 (43.4%)	14 (43.8%)	17 (77.3%)

Thank you for your effort in streamlining the results table. Based on feedback from 2 reviewers, we have split Table 2 into 2 tables - one with summarized results similar to that suggested here (new Table 2), and one showing the differences amongst sample types (Table 3).

Line 392: "Figure 3" should be "Figure 5".

Thank you; this has been fixed.

Figure 5: Please add the sequence lengths of the individual gene regions (*cob, cox1, nad2*), as well as the total length of the concatenated sequences used for the construction of the haplotype network.

Thank you for this suggestion. We have added sequence lengths of the individual gene regions and the total concatenated sequence for our haplotype in the "Molecular analysis (Sanger sequencing)" section of the Methods. We also added the concatenated sequence length used for the haplotype analysis to the Figure 5 caption. All of the sequences are also available in a .fasta file in S4.

Discussion:

Lines 436-438: "…since at least 2021" – Why is this particular year mentioned? Is there reason to believe this transmission cycle was established this recently?

We began our scat and carcass collection in 2021, so that is when the earliest known infected sample was collected. We have changed the sentence to reflect this (and added the years to the methods). It is likely that coyotes in the region have been infected for much longer, but we cannot know with certainty.

Lines 485-489: As a note from personal experience, it is rather unlikely that *Taenia* eggs are more resistant to freeze-thaw cycles than those of *Echinococcus*.

We wonder if the reviewer meant "it is rather unlikely that *Taenia* eggs are less resistant," i.e., *Taenia* eggs are more likely to crack than those of *Echinococcus*? This would reflect feedback from another reviewer, and we have added a sentence to that end: *"E. multilocularis is present as far north as the Arctic region, while Taenia pisiformis (the other taeniid species we detected) is a subarctic parasite, which might explain why E. multilocularis eggs are more freeze-resistant."* (lines 474-476)

Line 550: "(Massolo et al., 2014)" should be "(1)".

Thank you for catching this citation error; we have updated it accordingly.

Lines 564-566: This is perhaps a misunderstanding. Muskrats are not thought to be the ecologically most important intermediate hosts in Europe, where they are an invasive species. They exhibit the highest prevalence for *E. m.*; however, muskrats are restricted to only a few habitats in Europe, and they are not as common and widespread (and a part of the diet of foxes) as, for example, voles.

Thank you for this clarification. In accordance with another reviewer's feedback to shorten the discussion, we have removed this sentence.
