# Supplementary material for: Detection of Echinococcus multilocularis in coyotes in Washington State, USA highlights need for increased wildlife surveillance
Source: PLoS Negl Trop Dis. 2026 Mar 24;20(3):e0013502. doi: 10.1371/journal.pntd.0013502 (PMC13012483; doi:10.1371/journal.pntd.0013502)
Supplement: Attachment — Submitted filename: Review_Hentati et al 2025.docx [file pntd.0013502.s012.docx]

This nicely written manuscript provides new data on the presence and spread of *E. multilocularis* in coyotes in Washington State (USA) and highlights the implications for alveolar echinococcosis risk in the Pacific Northwest.

Gastrointestinal tracts obtained from coyote carcasses, as well as field-collected scats were examined for *Echinococcus* worms and eggs. DNA was extracted from faecal samples, intestinal swabs, and tapeworm material for molecular analysis and *E. multilocularis* haplotype identification. Individual coyotes were identified using microsatellite markers. As a byproduct, the sensitivity of morphological and molecular detection methods was evaluated and compared.

In total, almost half (45.7%) of the 81 examined coyote carcasses were infected with *E. multilocularis*. The *E. multilocularis* haplotype identified in four individual coyotes was previously reported from British Columbia (Canada) and is closely related to haplotypes from France. None of the 30 field-collected scat samples tested positive for *E. multilocularis*, which might be due to a methodical error. This is briefly discussed.

This manuscript is a proper piece of work which confirms that *E. multilocularis* has an established sylvatic life cycle in Washington State. I have only few suggestions for minor amendments and corrections:

**Introduction:**

Figure 1: The light-yellow colouration of the endemic areas pre 1990s is barely distinguishable from the white background. Please choose a darker tint for better visibility. This is also the case for Figure 2.

Line 70: Missing parenthesis – “(Figure 1; (10-18))”.

Lines 120-122: It might be interesting to deliberate (either here or in the discussion) if the increase of human AE cases in North America can also be linked to an increased awareness of this disease among health professionals (fewer misdiagnoses).

**Methods:**

Lines 182-200: The segments 'Potential definitive hosts' and 'Potential intermediate hosts' do not belong in the 'Methods' chapter. As results of a previous study, information on the diet of Washington coyotes may be included as part of the introduction (possible wildlife hosts in Washington) or in the discussion ('Transmission dynamics…').

Line 193: Period after “(50)” should be a comma.

Lines 201-207: In which years were the carcasses collected? Please add this information.

Line 235: The G in “Gloves” is missing.

Lines 272-274: The “freshness categories” should be mentioned in the segment 'Coyote scat collection' rather than here.

Lines 310-315: Why was the haplotype analysis limited to only six positive coyotes? Is there a reason why it was it necessary to confirm *E. multilocularis* both morphologically and with Illumina sequencing before Sanger sequencing? Worms were present in 33 individuals – why not genetically identify them with Sanger sequencing?

**Results:**

The field-collected scat samples were not screened for the presence of taeniid eggs via flotation, correct? It would have been interesting to note if *T. pisiformis* was detected in all taeniid-positive samples or if other taeniid species/ low egg counts were missed by the NGS approach.

Table 2: Separating the scat results into their “freshness categories” in Table 2 seems rather unnecessary, as *E. multilocularis* was not detected in any of them and the freshness aspect is not further discussed (for example regarding successful *Taenia* detection). It might also make more sense to change the table setup to something like the following, for clarity:

|  |  | Available sample type | | |
| --- | --- | --- | --- | --- |
| Coyote carcass samples | total | Morphological ID | Faecal samples | Intestinal swabs |
| n dissected individuals | 81 | 76 | 32 | 22 |
| *E.m.* positive | 37 (45.7%) | 33 (43.4%) | 14 (43.8%) | 17 (77.3%) |

Line 392: “Figure 3” should be “Figure 5”.

Figure 5: Please add the sequence lengths of the individual gene regions (*cob, cox1, nad2*), as well as the total length of the concatenated sequences used for the construction of the haplotype network.

**Discussion:**

Lines 436-438: “…since at least 2021” – Why is this particular year mentioned? Is there reason to believe this transmission cycle was established this recently?

Lines 485-489: As a note from personal experience, it is rather unlikely that *Taenia* eggs are more resistant to freeze-thaw cycles than those of *Echinococcus*.

Line 550: “(Massolo et al., 2014)” should be “(1)”.

Lines 564-566: This is perhaps a misunderstanding. Muskrats are not thought to be the ecologically most important intermediate hosts in Europe, where they are an invasive species. They exhibit the highest prevalence for *E. m*.; however, muskrats are restricted to only a few habitats in Europe, and they are not as common and widespread (and a part of the diet of foxes) as, for example, voles.
